# A Deep Neural Network for Identifying DNA N4-Methylcytosine Sites

**DOI:** 10.3389/fgene.2020.00209

**Published:** 2020-03-06

**Authors:** Feng Zeng, Guanyun Fang, Lan Yao

**Affiliations:** ^1^School of Computer Science and Engineering, Central South University, Changsha, China; ^2^College of Mathematics and Econometrics, Hunan University, Changsha, China

**Keywords:** N4-methylcytosine, machine learning, deep neural network, CNN, BLSTM, integrated algorithm

## Abstract

**Motivation:** N4-methylcytosine (4mC) plays an important role in host defense and transcriptional regulation. Accurate identification of 4mc sites provides a more comprehensive understanding of its biological effects. At present, the traditional machine learning algorithms are used in the research on 4mC sites prediction, but the complexity of the algorithms is relatively high, which is not suitable for the processing of large data sets, and the accuracy of prediction needs to be improved. Therefore, it is necessary to develop a new and effective method to accurately identify 4mC sites.

**Results:** In this work, we found a large number of 4mC sites and non 4mC sites of *Caenorhabditis elegans* (*C. elegans*) from the latest MethSMRT website, which greatly expanded the dataset of *C. elegans*, and developed a hybrid deep neural network framework named 4mcDeep-CBI, aiming to identify 4mC sites. In order to obtain the high latitude information of the feature, we input the preliminary extracted features into the Convolutional Neural Network (CNN) and Bidirectional Long Short Term Memory network (BLSTM) to generate advanced features. Taking the advanced features as algorithm input, we have proposed an integrated algorithm to improve feature representation. Experimental results on large new dataset show that the proposed predictor is able to achieve generally better performance in identifying 4mC sites as compared to the state-of-art predictor. Notably, this is the first study of identifying 4mC sites using deep neural network. Moreover, our model runs much faster than the state-of-art predictor.

## 1. Introduction

DNA methylation is a form of chemical modification of DNA, which alters genetic performance without altering the DNA sequence. Numerous studies have shown that DNA methylation can cause changes in chromatin structure, DNA conformation, DNA stability, and DNA-protein interactions, thereby controlling gene expression (Wang and Qiu, 2012). In many species, the N-methylation would inhibit Watson-Crick hydrogen bond formation with guanosine (Fazakerley et al., 1987). The differential susceptibility of foreign DNA and self-DNA suggests that some process, such as cytosine methylation, may be affording protection to nuclear DNA (Carpenter et al., 2012). DNA methylation guided by specific methyltransferase enzymes occurs in both prokaryotes and eukaryotes. These modifications can label genomic regions to control various processes including base pairing, duplex stability, replication, repair, transcription, nucleosome localization, X chromosome inactivation, imprinting and epigenetic memory (Iyer et al., 2011; Allis and Jenuwein, 2016; O'Brown and Greer, 2016). The most widespread DNA methylation modifications are N6-methyladenine (6mA), 5-methylcytosine (5mC) and N4-methylcytosine (4mC) that have been detected in both prokaryotic and eukaryotic genomes (Fu et al., 2015; Blow et al., 2016; Chen et al., 2017). These modifications are catalyzed by specific DNA methyltransferases (DNMTs) that transfer a methyl group to specific exocyclic amino groups (He et al., 2018). In eukaryotes, 5mC is the most common DNA modification, which is essential for gene regulation, transposon suppression and gene imprinting (Suzuki and Bird, 2008). While 6mA and 4mC are very small, they can only be detected in eukaryotes by high sensitivity techniques. In prokaryotes, 6mA and 4mC are the majority, mainly used to distinguish host DNA from exogenous pathogenic DNA (Heyn and Esteller, 2015), and 4mc controls DNA replication and corrects DNA replication errors (Cheng et al., 1995; Wei et al., 2018). Moreover, 4mC as part of a restriction-modification (R-M) system prevents restriction enzymes from degrading host DNA (Schweizer et al., 2008; Wei et al., 2018).

Although extensive studies have been conducted on modifications of 5mC and 6ma, studies on 4mC are relatively limited due to the lack of effective experimental methods and large amounts of data. Single-molecule real-time sequencing (SMRT) technology can detect 4mC, 5mc, and 6mA base modifications (Ecker, 2010; Flusberg et al., 2010; Clark et al., 2013; Davis et al., 2013). However, SMRT sequencing is costly and is not conducive to the analysis of various species. Recently, Yu et al. (2015) proposed a method for the determination of methylcytosine in genomic DNA by 4 mC-Tet-assisted bisulfite sequencing, which can accurately generate a genome-wide, single-base resolution map of 4mC, and finally identify the 4mC motif associated with the bacterial R-M system. Biological experiments are laborious and expensive when performing genome-wide testing. Therefore, it is necessary to develop a calculation method for identifying 4mC sites.

So far, there are only four methods for identifying the 4mC sites, all of which adopt the SVM model, including iDNA4mC, 4mCPred, 4mcPred-SVM and 4mcPred-IFL. The four predictors are designed to predict 4mC sites directly from sequences. The first 4mC site predictor, called iDNA4mC (Chen et al., 2017), encodes DNA sequences using nucleotide chemistry properties and frequency and is tested across different species. The experimental results show that iDNA4mC has achieved initial results in identifying 4mC sites. However, the low predictive power is the main drawback of iDNA4mC. The second 4mC site predictor, called 4mCPred (He et al., 2018), proposes a new feature coding algorithm by combining position-specific trinucleotide propensity and electron-ion interaction pseudopotentials, which improves the accuracy of prediction. The third 4mC site predictor, called 4mcPred-SVM (Wei et al., 2018), proposes more useful sequence features in the predictor and improves the feature representation capability through a two-step feature selection method. However, the performance of the experiment did not improve much. Recently, Wei et al. (2019) proposed the fourth 4mC site predictor, called 4mcPred-IFL, which uses an iterative feature representation algorithm to learn probabilistic features from different sequential models and enhance feature representation in a supervised iterative manner. However, the complexity of 4mcPred-IFL is very high. When the data set is large, it takes a long time to obtain the results. Meanwhile, the prediction accuracy in 4mcPred-IFL can be improved further.

In this work, we developed a deep learning framework called 4mcDeep-CBI to identify the 4mC sites. Deep learning related methods are widely used in hot spots prediction of protein-protein interfaces (Pan et al., 2018; Wang et al., 2018; Deng et al., 2019; Liu et al., 2019), but we have not found any work with deep learning in 4mC sites prediction, and all previous studies have used SVM machine learning methods. This work is the first study of 4mC sites using deep learning. Especially, we have greatly expanded the dataset which is used to evaluated the prediction models of the 4mC sites. Experimental results demonstrate that 4mcDeep-CBI has better performance than other models. The contributions of our work can be summarized as follows.

(1) We have greatly expanded the dataset of *C. elegans*, and the number of samples was increased from 3,108 to 17,808, which is beneficial for subsequent research.
(2) we developed a deep learning framework to identify the 4mC sites. 3-CNN and BLSTM are used to extract deep information from the acquired features and to obtain advanced features. Experimental results show that advanced features have achieved better performance in identifying the 4mC sites.
(3) We finally take probability feature matrix obtained by the machine learning methods into the deep learning model, which further improve the prediction accuracy. In our experiment, compared with the state-of-art predictor, the proposed model has the accuracy increased from 87 to 93%.

## 2. Materials and Methods

### 2.1. Datasets

We obtained samples genomes of *Caenorhabditis elegans* (*C. elegans*) from the latest MethSMRT website, found a lot of 4mC sites and non 4mC sites with the sequence lengths all of 41 bp. Each 4mC sequence sample has several indicators: position, coverage, IPDRatio (inter-pulse duration ratio), frac, fracLow, fracUp, identificationQv. In order to construct a reliable quality dataset, we did the following two steps. Firstly, as stated in the Methylome Analysis Technical Note, the Modification QV (modQV) score indicates that the IPD ratio is significantly different from the expected background. Since the modQV score of 30 is the default threshold for calling a position as modified, we removed the sample with the modQV score more than 30. Secondly, as elaborated in previous study (Chou et al., 2015), if training and testing are conducted through this biased dataset, the experimental results may have overestimated accuracy. To eliminate redundancy and minimize the bias, the CD-HIT software (Fu et al., 2012) with the cut off threshold set at 80% was used to remove those sequences with high sequence similarity. After the above two steps, we obtained 15, 639 samples in *C. elegans*.

We combine the new samples with the *C. elegans* benchmark dataset (Ye et al., 2017) that was used in the previous works to form a new data set with 18, 747 samples. Some of the new samples we extracted may be similar to the previous benchmark dataset. Therefore, we use the CD-HIT software to remove those samples with high sequence similarity. Finally, we get the new *C. elegans* dataset with 17, 808 samples which contains 111, 73 positive samples and 663, 5 negative samples. The positive samples are the sequences centroided with functional 4mC sites detected by the SMRT sequencing technology, while the negative samples are the sequences with the cytosines in the center but not detected as 4mC (Wei et al., 2019). The new dataset can be downloaded from our github, and the download link is given in section 3.

### 2.2. Model of 4mcDeep-CBI

#### 2.2.1. Preliminary Feature Extraction

We use the eight features mentioned in Chen et al. (2017), He et al. (2018), Wei et al. (2018), and Wei et al. (2019) as preliminary features. These features are obtained by encoding the different sequence information by the feature representation algorithm of the sequence. These features are BKF (Binary and k-mer frequency), DBPF (Dinucleotide binary profile and frequency), KNN (K-Nearest Neighbor), PCP (Physical-Chemical Properties), MMI (Multivariate Mutual Information), PseDNC (Pseudo dinucleotide composition), PseEIIP (Electron-ion interaction pseudopotentials of trinucleotide) and RFHCP (Ring-function-hydrogen-chemical properties). The related feature extraction methods can be found in Wei et al. (2019).

#### 2.2.2. 4mcDeep-CBI Network

**Figure 1:**
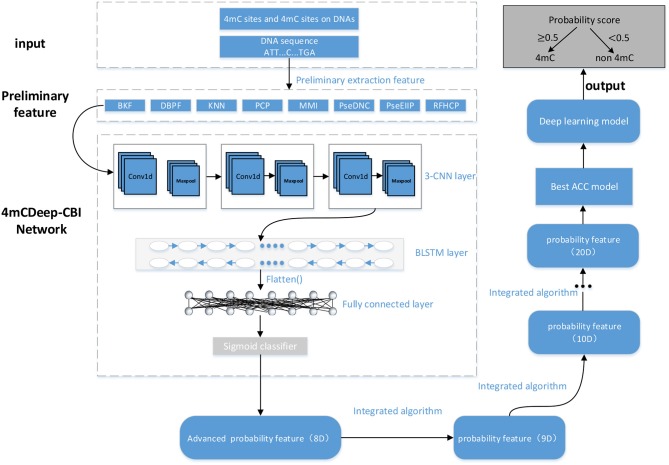
A graphical illustration of the 4mcDeep-CBI model.

As shown in Figure 1, 4mcDeep-CBI consists of 3-CNN layer, BLSTM layer, fully connected layer, and a sigmoid classifier. The input of 4mcDeep-CBI is one of eight preliminary features. First of all, the preliminary feature is used as the input to 3-CNN layer, which contains convolution layer, ReLU activation function and max pooling operation. Next, the output of 3-CNN layer will be imported to BLSTM layer to obtain an advanced feature. With the eight features as the inputs, we can get eight advanced features, respectively. Then, each advanced feature (matrix) will be further converted to one-dimensional feature (vector) using the flatten function, which will be finally connected to the fully connected layer. The last layer is the sigmoid layer, which is used to obtain advanced probability features and the prediction result of the first step. At last, we get an eight-dimensional feature, which will be the input of the integrated algorithm.

##### 2.2.2.1. Convolutional neural network (CNN)

CNN has a powerful ability to extract abstract features, which is not only suitable for image processing, but also for natural language processing tasks. It consists of convolution, activation, and max-pool layers.

In the model design, since we have verified in experiment that the model with 3 CNN layers has the best performance, we employ 3-CNN as an advanced feature extractor, and the input is the preliminary feature extracted from DNA sequences. We first put the preliminary features into the 3-CNN layer, respectively, and set the weighting parameters of the convolution filter. Then, the convolution layer outputs the matrix inner product between the input preliminary feature and filters. After convolution, a rectified linear unit (ReLU) is applied to sparsify the output of the convolution layer. The Rectified Linear Unit (ReLU) (Nair et al., 2010) takes the output of a convolution layer and clamps all the negative values to zero to introduce non-linearity that can not only reduce the computational cost, but also avoid the phenomenon of vanishing gradient and over-fitting. Finally, a max pooling operation is used to reduce the dimensionality and over-fitting by taking the maximum value in a fixed-size sliding window. The output of the convolution module is represented by the following expression:

$$O_{c}=Pool\left(ReLU\left(Conv\left(S\right)\right)\right),$$

where *O*_*c*_ is the output tensor, *S* is the input preliminary feature of the sequence. For BKF as an example, the dimension of *S* is 1 × 500 × 1 (input_shape). The nb_filter of 3-CNN are 16, 32, 64, respectively, and the filter_length of 3-CNN are all 8. The parameters of max pool is 2. Therefore, the dimension of *O*_*c*_ is 1 × 223 × 64.

##### 2.2.2.2. Long short term memory networks (LSTM)

LSTM is a recurrent neural network (RNN) architecture (an artificial neural network) published in 1997 (Hochreiter and Schmidhuber, 1997). Compered with traditional RNNs, LSTM network is well-suited to learn from experience to classify, process and predict time series, and it has advantages in dealing with long term dependency. Especially, Bidirectional LSTM can capture the bidirectional dependence of features and the outputs of individual directions are concatenated, which can well mine the deeper information in the features:

$$O_{r}=BiLSTM\text{(}O_{c}\text{)},$$

where *O*_*r*_ is the output of BLSTM layer and is also advanced feature of the sequence, *O*_*c*_ is the feature matrix of a sequence obtained by the 3-CNN layer. A LSTM contains a forget gate layer, an input gate layer and an output gate layer. When the LSTM traverses each element of the input, it first determines what information the forget gate layer is about to discard based on the previous input. The input gate layer then determines what information should be stored for the next layer and updates the current state value. Finally, the output gate layer will only output the part of our output that we determined (Pan and Shen, 2018).

### 2.3. Integrated Algorithm Model

In the integrated algorithm model, there are six machine learning algorithms involved, which are K-nearest neighbor algorithm, Logistic regression algorithm, Support vector machine algorithm, Naive Bayesian algorithm, Decision tree algorithm, and Random forest algorithm, respectively. With the 8-D advanced feature of the sequence as the input, we run these six different machine learning algorithms to predict the labels, and get the best result. Then, the obtained probability value is combined with the previous 8-D advanced feature vector to form a new 9-D feature vector. Next, the 9-D feature are imported into the integrated algorithm model for the new iteration. This process will be repeated until performance reaches convergence. In each iteration, the multi-dimensional input features are trained, and the optimal algorithm is selected each time to obtain an one-dimensional probability feature, and then the input and output features are merged into a new feature vector which has one more dimension than the input and will be the new input for next iteration. For example, it is supposed that the vectors *f*_1_, *f*_2_, …, *f*_8_ are the advanced features obtained by previous processing, and with (*f*_1_, *f*_2_, …, *f*_8_) as the algorithm input, we can get the result vector *f*_9_. Then, (*f*_1_, *f*_2_, …, *f*_8_, *f*_9_) will be the algorithm input of the next iteration. If there are 5 iterations, we will get the result (*f*_1_, *f*_2_, …, *f*_8_, *f*_9_, *f*_10_, *f*_11_, *f*_12_, *f*_13_) which will be the feature matrix for the following processing. In the experiment, after less than 10 iterations, the algorithm can reach the state of convergence, which can be shown in section 3.

### 2.4. Deep Learning Model

For the last part of 4mcDeep-CBI, a general neural network model is used to get the optimal solution. The neural network has 2–4 intermediate layers, each with a different activation function. In our experiment, we used two layers of intermediate layers, each using the ReLU function as the activation function, and finally used the sigmoid function as the output layer. We found that inputting the advanced feature matrix obtained by the integrated algorithm into the neural network model can further improve the accuracy.

### 2.5. Performance Evaluation

For performance evaluation, we used the following five generally-used metrics: Sensitivity (SN), Specificity (SP), Accuracy (ACC), Mathew's Correlation Coefficient (MCC) (Wei et al., 2019) and Area Under the ROC Curve (AUC). The definition of each evaluation metric is as follows:

$$\begin{array}{l} \;SN=\frac{TP}{TP+FN},\\ \;SP=\frac{TN}{TN+FP},\\ \;ACC=\frac{TP+TN}{TP+TN+FN+FP},\\ MCC=\frac{TP*TN-FP*FN}{\sqrt{\left(TP+FN\right)\left(TP+FP\right)\left(TN+FP\right)\left(TN+FN\right)}}, \end{array}$$

where TP indicates that the actual result is a positive sample, and the predicted result is also a positive sample; TN indicates that the actual result is a negative sample, and the predicted result is also a negative sample; FP indicates that the actual result is a negative sample, and the predicted result is a positive sample (indicating that the negative sample is predicted incorrectly); FN indicates that the actual result is a positive sample, and the prediction result is a negative sample (indicating that the positive sample is predicted incorrectly).

The area under the ROC curve (AUC) is a comprehensive used metric. The abscissa of the ROC curve is the false positive rate and the ordinate is the positive rate. The AUC value is the enclosed area value of the ROC curve and the coordinate axis, and the value is between 0 and 1. The maximum value of AUC is 1, which means that the performance of the model is perfect, and all prediction results are correct. AUC value of 0 means that the model performance is very poor, and all prediction results are wrong.

## 3. Result and Discussion

We have done extensive experiments on the new dataset using the proposed predictor (4mcDeep-CBI) and the state-of-art predictor (4mcPred-IFL), respectively, then we make a performance comparison between two models. The dataset and code used in the experiment have been uploaded to our GitHub (https://github.com/mat310/4mcDeep), which is shared with other researchers. Due to limited space, part of experimental results are listed in Supplementary Material.

### 3.1. Performance of Different Features Used in Prediction

**Figure 2:**
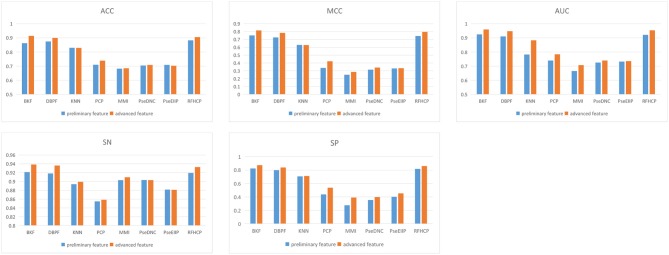
Evaluate the performance of preliminary feature and advanced feature on the same data set.

We put 8 preliminary features into the 3-CNN and BLSTM models to obtain advanced features. Then the advanced feature are sequentially passed through sigmoid classifier to obtain the prediction result of the first step. We performed different types of features for predictive performance analysis and compared the experimental results of 4mcPred-IFL with 4mcDeep-CBI. From Figure 2, we find that the predicted performance of the four features BKF, DBPF, KNN, and RFHCP ranks in the top four in the experimental results of both modes. In addition, the performance metrics of the eight characteristic experimental results have been improved in our model (The experimental results can be found in Tables S1, S2). Figure 2 shows that our proposed model performs better than 4mcPred-IFL in the preliminary experimental results.

**Figure 3:**
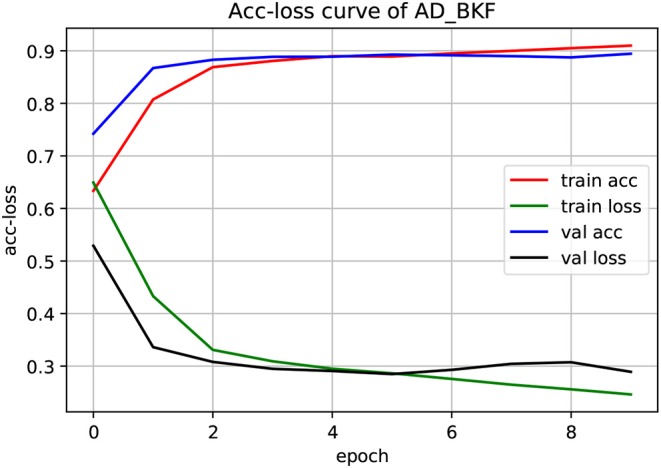
Acc-loss curve of AD_BKF based on 3-CNN and BLSTM models. Where AD_BKF is a advanced feature of BKF.

The experiment used a three-fold cross-validation. As shown in Figure 3, this is the acc-loss curve of AD_BKF during the preliminary experiment (acc-loss curves of other advanced feature can be found in Figure S1). Epoch refers to the number of times when all data were sent into the network to complete one forward calculation and back propagation. As can be seen from the figure, with the increase of epoch value, the accuracy of the training set and verification set increased continuously, and finally converged at epoch = 5. The loss function values of the training set and verification set decreased continuously, and finally converged when epoch = 5. Therefore, we can set epoch = 5 to get the best experimental results. Figure 3 illustrates that the prediction performance is continuously improved and there is no over-fitting during the experiment.

### 3.2. Performance of the Integrated Algorithm

**Figure 4:**
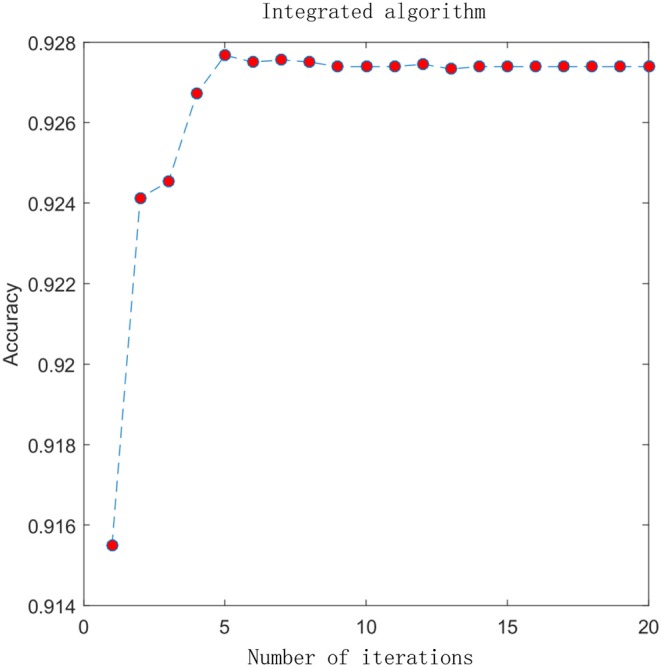
Experimental result graph after using integrated algorithm.

In the previous section, we compared the experimental results of different advanced features. Here, we combine the advanced probability features obtained from the sigmoid classifier to form a matrix with 8-D probabilistic feature. This matrix is input into the integrated algorithm model and we get the experimental results. To visually analyze the results, we plot the ACC change with the increment of the feature size, which is shown in Figure 4. In the figure, the X-axis represents the number of iterations and the Y-axis represents the performance in terms of accuracy. Before performing the iterative operation, we have a matrix with 8-D probabilistic feature. As the number of iterations increases, performance increases rapidly from the beginning, reaching a maximum after 5 iterations when the feature size of the matrix is 13 and ACC is 0.9274, then gradually converge to a steady state. This suggests that the integrated algorithm model can improve feature representation and surely improve performance. 4mcPred-IFL adopted an iterative feature representation algorithm, which reached the maximum when the number of iterations was 30 and ACC was 0.9001, and then gradually converges to a stable state. The details can be found in Figure S2.

### 3.3. 4mcDeep-CBI vs. State-of-Art Predictor on Performance

**Figure 5:**
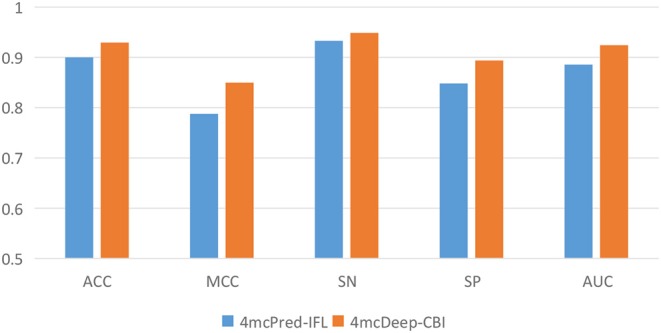
Performance evaluation of our predictor and the state-of-the-art predictor on the same dataset.

**Figure 6:**
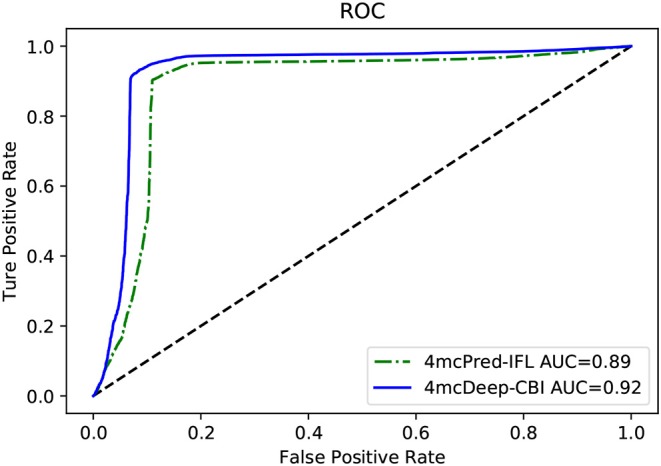
ROC curves of our predictor and the state-of-the-art predictor on the same dataset.

Our 4mcDeep-CBI model shows the best predictive performance, and we achieve ACC = 0.9294, MCC = 0.8498, SN = 0.9486, SP = 0.8938, AUC = 0.9242. To further evaluate the performance of our predictor 4mcDeep-CBI, we compared our predictor with the state-of-art predictor: 4mcPred-IFL. The performances of 4mcDeep-CBI and 4mcPred-IFL are depicted in Figures 5, 6, respectively. Figure 5 illustrates the performances in terms of ACC, MCC, SN, SP, and AUC, while Figure 6 shows the ROC curves of 4mcDeep-CBI and 4mcPred-IFL. The details of their performances can be found in Table S3. It can be clearly seen that 4mcDeep-CBI achieved better performance than 4mcPred-IFL in all five metrics. Our predictor improves ACC by 3.26%. It is worth noting that our predictor increased the MCC by 7.88%. MCC is essentially a correlation coefficient between the actual classification and the prediction classification, and is a relatively comprehensive metric. This shows that 4mcDeep-CBI is better than 4mcPred-IFL in terms of comprehensiveness and integrity.

The ROC curve between the different methods is shown in Figure 6. As can be seen from the figure, the ROC curve of 4mcDeep-CBI is closer to the upper left corner, and the area under the ROC curve is the largest, which is 4.35% larger than that of 4mcPred-IFL. In summary, the above results illustrate that the performance of 4mcDeep-CBI is better than 4mcPred-IFL, and 4mcDeep-CBI can effectively improve the accuracy of identifying 4mC sites.

### 3.4. 4mcDeep-CBI vs. State-of-Art Predictor on Running Time

The running time of the main modules of 4mcPred-IFL and 4mcDeep-CBI accounts for a large proportion in their respective models. Among them, the main module of 4mcPred-IFL refers to the preliminary experimental results obtained by putting the extracted preliminary features into the SVM model. The main module of the 4mcDeep-CBI model refers to the preliminary experimental results obtained by putting the extracted preliminary features into the deep learning model. In order to explore the operational efficiency of the model, we run the main modules of 4mcPred-IFL and 4mcDeep-CBI separately on the same server. The preliminary feature is BKF as an example. Experiments are carried out with different sample sizes. The results obtained are shown in Table 1. 4mcPred-IFL employed Sequential Forward Search (SFS) to determine the optimal feature subset. In Table 1, “SVM_10” refers to the distance of the SFS is 10, and “SVM_50” refers to the distance of the SFS is 50. The smaller the distance setting, the greater the possibility of better experimental results, and the longer the experiment runs. In addition, when the distance range from 10 to 50, the optimal subset of features can be obtained. As we can see in Table 1, our model runs much faster than the state-of-art predictor. After running 16, 000 samples, 4mcDeep-CBI need 48.2 min only, but even if the distance is set to 50, 4mcPred-IFL takes 3261.3 min to run. The running time is more than 50 times slower than us. Moreover, as the number of samples increased, 4mcDeep-CBI grew more slowly than 4mcPred-IFL. There are at least two reasons: (1) The efficiency of 4mcpred-IFL using SFS method to obtain the optimal feature set is very slow. (2) There are two important parameters (the penalty parameter *C* and the kernel parameter γ) in the SVM model used by 4mcPred-IFL. Meanwhile, 4mcPred-IFL takes a lot of time to call SVM algorithm over and over again to optimize the penalty parameter *C* and the kernel parameter γ by using the grid search method. Consequently, the complexity of the 4mcpred-IFL model is much higher than our proposed model.

**Table 1:** Running time of the main modules of 4mcPred-IFL and 4mcDeep-CBI.

	**Running_time (minute)**		
**Sample size**	**SVM_10**	**SVM_50**	**4mcDeep-CBI**
1,000	31.3	9.2	3.1
4,000	1034.4	222.1	10.7
7,000	3123.6	698.4	19.8
10,000	6255.8	1365.6	24.5
13,000	9449.5	2173.2	35.1
16,000	15094.4	3261.3	48.2

### 3.5. Impact of Different CNN Layers on 4mcDeep-CBI

**Figure 7:**
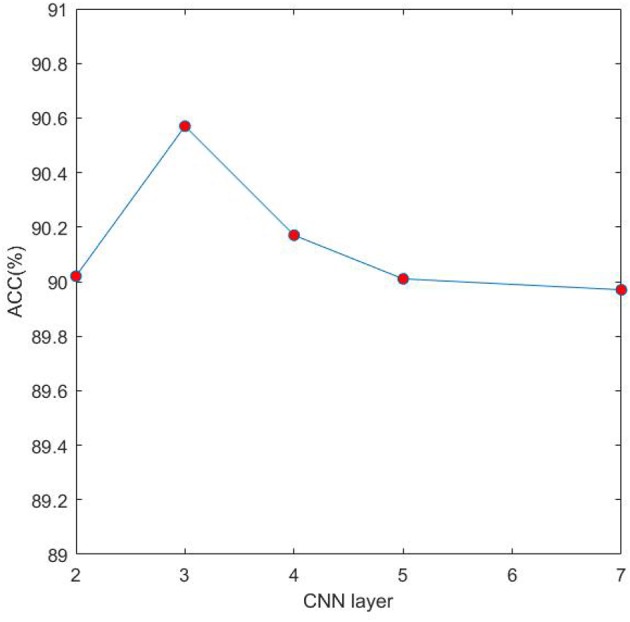
Impact of different CNN layers on ACC.

In the proposed model 4mcDeep-CBI, we have three CNN layers which can efficiently extract the features from input data. In the experiment, with the CNN layers given, we obtain the accuracy of the 4mcDeep-CBI, and we make a performance comparison according to different CNN layers. For feature RFHCP, Table 2 shows the experimental results of the 4mcDeep-CBI with 4 CNN layers. Parameters are set as batch_size = 32, 64, 128, 256; maxpool1D = 1, 2, 3; learning rate = 0.001, 0.005, 0.0001; dropout ratio = 0.1, 0.2, 0.5. It can be found from Table 2 that the maximum ACC value is 90.17% when the 4mcDeep-CBI has 4 CNN layers. Similarly, we do experiments based on different (2, 3, 5, and 7) CNN layers. The experimental results are shown in Figure 7. As can be seen from Figure 7, maximum ACC value is 90.57% when the 4mcDeep-CBI has 3 CNN layers. For other features, the experiment has the same result. Therefore, the experiment verifies that 3-CNN layer model has the best performance, that is why we choose 3 CNN layers in the model design of the 4mcDeep-CBI.

**Table 2:** ACC of 4mcDeep-CBI with 4 CNN layers under different parameters.

**nb_filter**	**Filter_length**	**ACC (%)**
4, 8, 16, 32	4, 4, 4, 4	90.02
4, 8, 16, 32	8, 8, 8, 8	89.46
4, 8, 16, 32	16, 16, 16, 16	88.70
8, 16, 32, 64	4, 4, 4, 4	90.17
8, 16, 32, 64	8, 8, 8, 8	90.02
8, 16, 32, 64	16, 16, 16, 16	89.25
16, 32, 64, 128	4, 4, 4, 4	89.78
16, 32, 64, 128	8, 8, 8, 8	89.37
16, 32, 64, 128	16, 16, 16, 16	89.18
32, 16, 8, 4	4, 4, 4, 4	89.36
32, 16, 8, 4	8, 8, 8, 8	89.29
32, 16, 8, 4	16, 16, 16, 16	88.31
64, 32, 16, 8	4, 4, 4, 4	89.89
64, 32, 16, 8	8, 8, 8, 8	88.72
64, 32, 16, 8	16, 16, 16, 16	87.97
128, 64, 32, 16	4, 4, 4, 4	90.03
128, 64, 32, 16	8, 8, 8, 8	89.96
128, 64, 32, 16	16, 16, 16, 16	89.09

## 4. Conclusion

In this paper, we propose a deep neural network named 4mcDeep-CBI, which can further boost the performance of identifying 4mC sites. Moreover, we found a large number of 4mC sites and non 4mC sites of *C. elegans* from the latest MethSMRT website, which greatly expanded the data set of *C. elegans*. The proposed model 4mcDeep-CBI uses 3-CNN and BLSTM modules to mine deep information of features to obtain advanced features. By experimental comparison with the state-of-art predictor, we found that our proposed framework performed better than the state-of-art predictor, and our model did not appear to have an over-fitting phenomenon. In addition, we have proposed an integrated algorithm to generate informative features. By analyzing the accuracy of the model during the iterative process, we find that the integrated algorithm is constantly improving the performance of the model. Finally, we evaluated our proposed 4mcDeep-CBI with the state-of-art predictor, and the results demonstrate that our model can achieve better performance in identifying 4mC sites and runs more efficiently. We hope that 4mcDeep-CBI can be an useful bioinformatics tool for identifying 4mC sites and promoting the DNA methylation analysis.

Deep learning is an important way of sequence analysis. For feature selection, we can use the most popular word embedding training method: Word2Vec algorithm, which can be combined with the secondary structure of DNA to predict 4mC sites. Moreover, the sequence length provided by the MethSMRT website is 41 bp, and we need longer DNA sequence fragments, such as 80, 100, and 150 bp to do further research.

## Data Availability Statement

The dataset and code used in the experiment have been uploaded to our GitHub (https://github.com/mat310/4mcDeep).

## Author Contributions

FZ and GF design the model, experiments, and wrote the paper. GF performed the experiments. LY analyzed the data, provided the suggestions to improve the performance, and contributed the materials and analysis tools.

### Conflict of Interest

The authors declare that the research was conducted in the absence of any commercial or financial relationships that could be construed as a potential conflict of interest.
