# Supplementary material for: A Deep Neural Network for Identifying DNA N4-Methylcytosine Sites
Source: Front Genet. 2020 Mar 6;11:209. doi: 10.3389/fgene.2020.00209 (PMC7067889; doi:10.3389/fgene.2020.00209)
Supplement: Supplementary file 1 [file Data_Sheet_1.PDF]

Supplementary Material

A deep neural network for identifying DNA N4-methylcytosine sites

Feng Zeng*, Guanyun Fang, Lan Yao*

*** Correspondence:** Feng Zeng and Lan Yao: fengzeng@csu.edu.cn and yao@hnu.edu.cn

Supplementary Tables

Table S1. The first experimental results of the 4mcPred-IFL using the preliminary function.

Feature	ACC	MCC	AUC	SN	SP
BKF	0.8623	0.7521	0.9251	0.9213	0.8244
DBPF	0.8748	0.7264	0.9107	0.9181	0.7985
KNN	0.8303	0.6306	0.7822	0.8939	0.7062
PCP	0.7099	0.3374	0.7394	0.8548	0.4368
MMI	0.6821	0.2499	0.6651	0.9130	0.2752
PseDNC	0.7043	0.3151	0.7253	0.9033	0.3537
PseEIIP	0.7087	0.3306	0.7320	0.8815	0.4014
RFHCP	0.8826	0.7441	0.9218	0.9193	0.8179

Table S2. The first experimental results of the 4mcDeep-CBI using the advanced function.

Feature	ACC	MCC	AUC	SN	SP
AD_BKF	0.9144	0.8161	0.9599	0.9385	0.8737
AD_DBPF	0.9	0.7845	0.9478	0.9362	0.839
AD_KNN	0.8293	0.6289	0.8828	0.8992	0.7117
AD_PCP	0.7387	0.4219	0.7837	0.8585	0.537
AD_MMI	0.6849	0.2859	0.7073	0.9096	0.3907
AD_PseDNC	0.7084	0.3419	0.7396	0.9031	0.3974
AD_PseEIIP	0.7027	0.334	0.7355	0.881	0.4529
AD_RFHCP	0.9057	0.7973	0.9541	0.9327	0.8601

Table S3. Comparison of the final experimental results of 4mcPred-IFLand4mcDeep-CBI.

**metric**	**4mcPred-IFL**	**4mcDeep-CBI**
ACC	0.9001	0.9294
MCC	0.7877	0.8498
SN	0.9329	0.9486
SP	0.8482	0.8938
AUC	0.8857	0.9242

Supplementary Figures

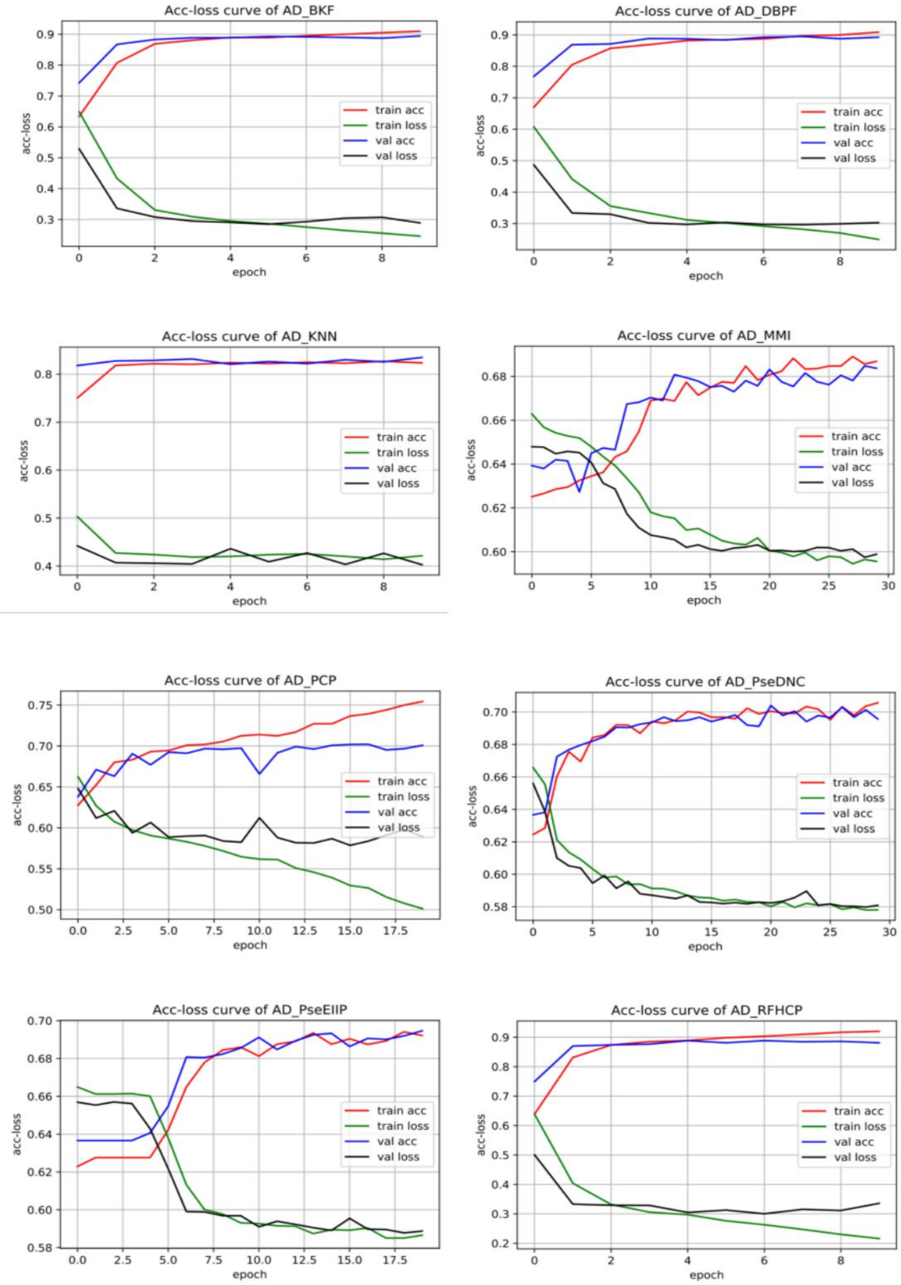

Figure S1. The acc-loss curve of advanced features based on CNN and BLSTM models

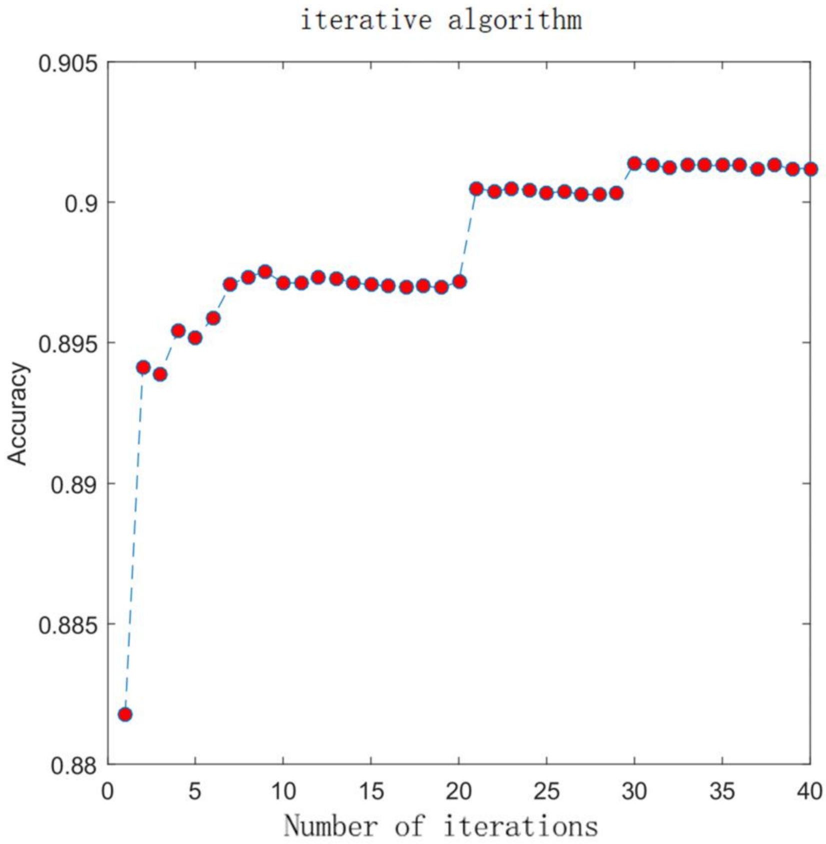

Figure S2. Experimental result graph of 4mcPred-IFL using iterative algorithm
